# On Behind the Physics of the Thermoelectricity of Topological Insulators

**DOI:** 10.1038/s41598-019-42744-3

**Published:** 2019-04-19

**Authors:** Daniel Baldomir, Daniel Faílde

**Affiliations:** Departamento de Física Aplicada, Instituto de Investigacións Tecnolóxicas. Universidade de Santiago de Compostela, E-15782 Campus Vida s/n, Santiago de Compostela, Spain

**Keywords:** Topological insulators, Theoretical physics

## Abstract

Topological Insulators are the best thermoelectric materials involving a sophisticated physics beyond their solid state and electronic structure. We show that exists a topological contribution to the thermoelectric effect that arises between topological and thermal quantum field theories applied at very low energies. This formalism provides us with a quantized topological mass proportional to the temperature T leading, through an electric potential V, to a Seebeck coefficient where we identify an anomalous contribution that can be associated to the creation of real electron-hole Schwinger’s pairs close to the topological bands. Finally, we find a general expression for the dimensionless figure of merit of these topological materials, considering only the electronic contribution, getting a value of 2.73 that is applicable to the Bi_2_Te_3_, for which it was reported a value of 2.4 after reducing its phononic contribution, using only the most basic topological numbers (0 or 1).

## Introduction

Nowadays topological insulators (TI) are the best thermoelectrics (TE) at room temperature^1–5^, specially if they are combined with nanotechnological structures able to reduce the phononic thermal conductivity. A good example is^6^ bismuth telluride, Bi_2_Te_3_, which has a small band gap giving a good number of carriers at room temperature (300 K) and reaching 2.4 for its dimensionless figure of merit ZT for p-type using alternating layers in a superlattice with Sb_2_Te_3_. This is the highest value of thermoelectricity observed^7^ so far at room temperature. Despite the fact that the electronic structure of these materials was exhaustively studied^8^ in relation with their thermoelectricity^9,10^, there is still lacking in the literature a physical model^11^ able to explicitly take into account their common topological and physical features. Its importance might appear obvious with a counterexample, Pb_1−*x*_Sn_*x*_Te has a good electronic structure to be a topological insulator with thermoelectricity^12^, but due to have an even number of band inversions, this prevents it to have time-reversal symmetry $\hat{T}$ and thus to be a TI, although it is a good thermoelectric at higher temperatures. Hence, the whole topology of a TI is not fully necessary for having good thermoelectricity as we are going to see. Understanding the physics behind these phenomena is not an easy task because it links different scientific branches which were developed independently: particle physics, statistical mechanics, condensed matter and algebraic topology. This is a characteristic of materials which exhibit linear dispersion laws instead of quadratic ones, allowing a quantum field interpretation where the spinors play a fundamental role substituting the usual non-relativistic wave function.

The paper is organized as follows. Firstly, we examine the basic concepts of topology and physics for topological insulators trying to show how they are related in the same structure. After that, we show that the Riemann-Hurwitz formula plays an important role which was not considered in the literature so far. This allows us to find five topological regions which are connected by four bands defined using the periodicity of the instanton solutions associated to the non-Abelian Berry fields introduced within the bulk of the TI. In the case of no time-reversal symmetry $\hat{T}$, we have only three topological regions, connected by two bands. Finally we present a straightforward relationship between the temperature T and the topological index *μ* with the scalar electric potential V. This leads to a Seebeck coefficient for which we identify two terms, one related to the topological electron pump, and another associated to a change in the topological index that might be associated to the creation of real electron-hole pairs as will be analysed. We end with the calculation of a general expression for the dimensionless figure of merit *ZT* of the edge states in TI, taking into account the electronic contribution and neglecting the phononic part^7,13^.

## Results

Solid state physics allow us to tackle the problem of a crystal with translation symmetry, reducing the analysis of its different physical properties to the first Brillouin zone. This is permitted thanks to Bloch theorem, i.e., for a periodic potential *V*(*x* + *a*) = *V*(*x*), where *a* is the spatial period or the lattice constant, the wave function associated to the electrons have also a periodicity *ψ*_*k*_(*x* + *a*) = *e*^*ika*^*ψ*_*k*_(*x*). Being the eigenvalues *ξ*_*k*_ = *ξ*_*k*_ + _*T*_, *ξ*_*k*_ = *ξ*_−*k*_ also periodic in time. These are the necessary conditions for calculating the energy bands in a solid. It is easy to see that parity $\hat{P}$, or space inversion is straightforwardly followed, while time-reversal symmetry $\hat{T}$ is not so obviously fulfilled. For example, the Schrödinger equation $i\hslash \frac{\partial }{\partial t}\psi (t)=H\psi (t)$ under time-reversal gives $i\hslash \frac{\partial }{\partial (-t)}\psi (\,-\,t)=H\psi (\,-\,t)$, where *ψ*(−*t*) is not a solution due to the first order time derivative. This can be solved if the $\hat{T}$ operator has also associated a complex conjugation $\hat{K}$ operator. In fact, we must define $\hat{T}=-\,i{\sigma }_{y}\hat{K}$ for spinless states where *U* is a unitary operator. In the case of having half-integer spin particles, the unitary operator can be written in function of the *σ*_*y*_ Pauli matrix as $U=exp(-i\frac{\pi }{2}{\sigma }_{y})$. This, given that ${\sigma }_{y}^{2}=1$, allow us to write the time-reversal operator as $\hat{T}=-\,i{\sigma }_{y}\hat{K}$, which acting on the multiparticle state gives *T*^2^ = 1 for an even number of fermions or *T*^2^ = −1 when the number is odd. More generally written, *T*^2^ has eigenvalue (−1)^2*s*^ for a particle of spin *s*, and if |*n*> is an energy eigenstate, then *T*|*n*> is also an eigenstate of the system, sharing the same energy and being orthogonal to each other. Thus, if there is an odd number of electrons there must be (at least) a twofold degeneracy; known as Kramers degeneracy. This is Kramers theorem, which when it is completed with the previous Bloch theorem for the bands, should provide us the first tools^14^ to examine topologically the first Brillouin Zone (BZ) of a TI.

**Figure 1:**
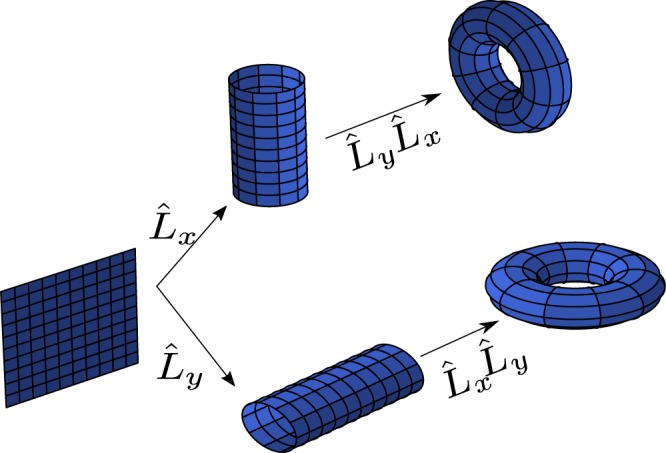
Evolution of the 2-D Brillouin Zone with trivial topology under different translation symmetries. Applying individual translation symmetries, two cylinders equivalent to two non-trivial topological *S*^1^ circles arise. Combining the two translations symmetries we obtain two equivalent torus *T*^2^ = *S*^1^ × *S*^1^.

In Fig. 1 we represent schematically how the 2D BZ evolves under different translation operations transforming the square BZ into two equivalent cylinders *S*_1_. Consequently, it is also easy to see that combining the two lattice periodicities available in two dimensions, a 2D torus *S*^1^ × *S*^1^ can arise. That is, the existence of translation symmetry allows us to transform a topologically trivial square into a non-trivial torus with genus *g* ≠ 0. Given that all crystals are able to develop these fundamental properties, non-trivial topology is not enough when we study TI and the presence of singularities which are usually counted on the band structure are needed. Obviously, without extra-information, these singularities break the translation symmetry or the periodicity of the Bloch states^15,16^. So, let’s analyze a little bit the possibility of these translations symmetry breaking. Physically the infinitesimal translations are generated by the linear momentum operator $\hat{L}(dx)=\hat{1}-\frac{i}{\hslash }\hat{p}(dx)$, where $[\hat{x},\hat{p}]=i\hslash \hat{1}$ follows the Heisenberg’s principle of uncertainty. By extending this operation over the whole crystal one gets the finite translation operator $\hat{L}=li{m}_{N\to \infty }{[\hat{1}-\frac{i}{\hslash }\hat{p}(\frac{a}{N})]}^{N}=\exp (-\frac{i}{\hslash }\hat{p}a)$. Taking into account the periodicity of the crystal potential *V*(*x*) = *V*(*x* + *a*), which involves the periodicity of the states, we can write *L*_*a*_|*ψ*(*x*)> = |*ψ*(*x*−*a*)> = exp(*ika*)|*ψ*(*x*)> where *k* is the wavelength number associated to the momentum *p* = ℏ*k*. On the contrary, the presence of singularities makes that |*ψ*(*x* − *a*)> = exp(*ika*)exp(*iγ*)|*ψ*(*x*)>, being *γ* the Berry phase which gives to electrons an anomalous phase factor during a complete cycle in the order parameter. Thus, in order to maintain the periodicity of the Bloch states, preserving the non-trivial topology that results for having a torus, singularities must take place on the edges of the crystal, where translation symmetry is no longer satisfied. Since singularities manifest in energy bands by means of Fermi points, bands are good hosts for feeding the non-trivial topology of a material whose electronic structure is appropriate and which its surface shows the breaking of translation symmetry. In this way, the Berry phase joints the non-trivial topology of the crystal employing its curvature and connection on the bands^17^.

### Riemann-Hurwitz formula and its application to topological insulators

The topological elements of the TI have been found, but now it remains to see how they work together employing their associated invariants. The Riemann-Hurwitz formula^18^, which generalizes the Euler topological invariant, enable us to construct an equation relating the genus g and g’ of two compact surfaces, i.e., whose boundary is zero. Actually this formula establishes the conditions for a map *f*:*T* → *S* being surjective and holomorphic, reducing the several topological invariants introducing the genus of *T*, the genus of *S*, *N* the degree of the map *f* and the number of ramifications *e*_*f*_(*k*). Riemann used the mentioned formula in the case that *S* were zero, i.e., spheres. Much later the general proofs were obtained by Zeuthen and Hurwitz. The formula is

1 $$\mathrm{2(}{g}_{T}-\mathrm{1)}=2N({g}_{S}-\mathrm{1)}+\sum _{k\in T}\,({e}_{f}(k)-\mathrm{1)}$$

**Figure 2:**
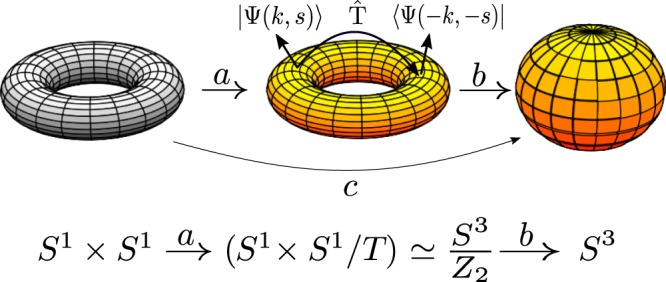
Schematic illustration of the projection of a torus into a sphere. The first torus is mapped by a on the second torus, which is equivalent to one sphere since it includes Kramers pairs with the same energy and orthonormal. The map b carries points of the previous torus on a real sphere. It is immediate to see that the map *c* = *b*⋅*a* is commutative.

In our case we have a 2D torus *T*^2^ with genus *g*_*T*_ = 1 mapped in a *S*^3^ sphere with genus *g*_*S*_ = 0 and the degree of the map *N* = 2 due to the Kramers double degeneracy, see Fig. 2. Hence we obtain five ramifications branches, i.e., *e*_*f*_(*k*) = 5. This can be directly related to the second Chern number, playing a fundamental role in the transformation of heat in electricity. The number of ramification branches diminishes to three when there are not Kramer pairs, that is, *N* = 1 with *e*_*f*_(*k*) = 3. But it is very remarkable to observe that this formula doesn’t depend on the dimensions of the involved sphere or torus, which justifies us to work with a *T*^2^ torus instead of a *T*^3^ or *T*^4^ without being worry about new results.

In the presence of singularities in the band structure the map c of Fig. 2 can be interpreted as the *d*_*a*_ map within the Hamiltonian *H* introduced^19^ to study TI in (4 + 1)-D,

2 $$H=\sum _{k}\,{\psi }_{k}^{+}{d}_{a}(k){{\rm{\Gamma }}}^{a}{\psi }_{k}$$

being ${d}_{a}(k)=(m+p{\sum }_{i}\,\cos \,{k}_{i},\,\sin \,{k}_{x},\,\sin \,{k}_{y},\,\sin \,{k}_{z},\,\sin \,{k}_{w})$ and Γ′*s* the Clifford matrices $\{{{\rm{\Gamma }}}^{\mu },{{\rm{\Gamma }}}^{\nu }\}=2{g}^{\mu \nu }{{\mathbb{1}}}_{5\times 5}$. We can calculate then, the second Chern number *C*_2_ associated to this Hamiltonian

3 $${C}_{2}=\frac{3}{8{\pi }^{2}}{\varepsilon }^{abcde}\int \,d{k}^{4}{\hat{d}}_{a}(k)\frac{\partial {\hat{d}}_{b}(k)}{\partial {k}_{x}}\frac{\partial {\hat{d}}_{c}(k)}{\partial {k}_{y}}\frac{\partial {\hat{d}}_{d}(k)}{\partial {k}_{z}}\frac{\partial {\hat{d}}_{e}(k)}{\partial {k}_{w}}$$

which is no more than the winding number resulting from the map ${\hat{d}}_{a}(k)\equiv \frac{{d}_{a}(k)}{|d(k)|}$ of a four-dimensional torus *T*^4^ to a sphere *S*^4^, having the mass *m* associated to the spinor’s Γ’*s* five critical values given by the condition ${\sum }_{k,\mu }{d}_{a}^{2}=0$ allowing us to identify, in the same way as the Riemann-Hurwitz theorem, five different branches for the second Chern number

4 $${C}_{2}\equiv \mu =\{\begin{array}{l}0,m\notin (\,-\,4p,4p)\\ +1,m\in (\,-\,4p,-\,2p)\\ -3,m\in (\,-\,2p,\,\mathrm{0)}\\ +3,m\in \mathrm{(0,}\,2p)\\ -1,m\in \mathrm{(2}p,\,4p)\end{array}$$

where *p* is taken as a mass parameter which must be equal to the background kinetic energy of the particles $2{k}_{B}T/{v}_{F}^{2}$ for keeping its physical meaning. In this way, *p* is mainly associated with the quantization of the temperature^20^. It is easy to see that for non-$\hat{T}$ symmetric Hamiltonians there are only three ramifications available for the second Chern number. As we saw, this can be interpreted, in one straightforward form, into the Riemann-Hurwitz formalism as two different maps, where we change the degree of the map N from 2 to 1.

### Topological Seebeck coefficient

Physically the non-Abelian Berry phase takes into account the allowed bulk degenerate states which are directly related with the change of temperature, as we shall see soon. First, we take the definition of the non-Abelian Berry connection ${a}_{j}^{\alpha \beta }(k)=i < \alpha ,{\bf{\text{k}}}|{\rm{\partial }}/{{\rm{\partial }}}_{{k}_{j}}|\beta ,{\bf{\text{k}}} >$ and the associated field (or curvature) ${f}_{ij}^{\alpha \beta }={\partial }_{i}{a}_{j}^{\alpha \beta }-{\partial }_{j}{a}_{i}^{\alpha \beta }+i{[{a}_{i},{a}_{j}]}^{\alpha \beta }$ and second Chern number ${C}_{2}=\frac{1}{32{\pi }^{2}}\int {d}^{4}k{\varepsilon }^{ijmn}tr({f}_{ij}{f}_{mn})$, where the indixes of the Levi-Civita tensor stand by *i*, *j*, *m*, *n* = 1, 2, 3, 4 and *α* refers to the occupied bands. This can be written within a pure gauge Yang-Mills formalism, where its solutions transform in general by ${a}_{\mu }={T}^{i}{a}_{\mu }^{i}$ and ${f}_{\alpha \beta }={T}^{i}{f}_{\alpha \beta }^{i}$ being the *T*^*i*^’*s* the generators of the inner symmetry group, which in our case will be *SU*(2)^21^. For a *U*∈*SU*(2) being position dependent, we have the gauge transformations for the potentials ${a}_{\alpha }\to {a}_{\alpha }^{U}={U}^{-1}{a}_{\alpha }U+{U}^{-1}{\partial }_{\alpha }U$ and the fields ${f}_{\alpha \beta }\to {f}_{\alpha \beta }^{U}={U}^{-1}{f}_{\alpha \beta }U$. It is immediate to observe that the non-Abelian fields are not gauge invariant, unlike what happens with the Abelian ones, which tell us that the curvature depends of the group representation but not the topological numbers. Thus, given that $tr({\varepsilon }^{ijmn}{f}_{ij}{f}_{mn})=4{\varepsilon }^{ijmn}{\partial }_{i}[tr({a}_{j}{\partial }_{m}{a}_{n}-\frac{2}{3}{a}_{j}{a}_{m}{a}_{n})]$, we can define the Chern-Simons term $W(a)=-\,\frac{\mu }{8{\pi }^{2}}\int {d}^{3}x{\varepsilon }^{ijm}tr({a}_{i}{\partial }_{j}{a}_{m}-\frac{2}{3}{a}_{i}{a}_{j}{a}_{m})$ where *μ* is a topological mass and which is not gauge invariant under gauge transformations

5 $$W({a}^{U})\to W(a)-\frac{\mu }{8{\pi }^{2}}\int \,{d}^{3}x[{\varepsilon }^{ijm}{\partial }_{i}tr({\partial }_{j}U{U}^{-1}{a}_{m})+\frac{\mu }{24{\pi }^{2}}\int \,{d}^{3}x{\varepsilon }^{ijm}tr({U}^{-1}{\partial }_{i}U{U}^{-1}{\partial }_{j}U{U}^{-1}{\partial }_{m}U)]$$

The first term of the integral is a total derivative which can be made zero in a manifold without boundary, while the second, is an integral written in short as *w*(*U*), which provide us topological information of the manifold as a winding number. This integral is actually an integer number coming from the homotopy group *π*_3_(*S*^3^) for the *SU*(2) group and we can take the Abelian Chern-Simons gauge symmetry associated to the background electrodynamics, i.e., the U(1) as a subgroup of transformations close to the surface. In this case, we can rewrite the Chern-Simons transformation for both fields. Hence the transformation of the Chern-Simons can be rewritten as

6 $$W(a)\to W(a)+\frac{\mu }{12\pi }24{\pi }^{2}w(U)=W(U)+2\pi \mu w(U)$$

But as the path integral ${e}^{i\frac{W}{\hslash }}$ must be invariant, hence it means that *μ* is an integer. These are going to be the dynamic fields meanwhile the electromagnetic gauge potential *A*_*α*_ will appear as a background gauge field.

Let us go now to the thermodynamic part of the topological thermoelectricity, applying the instanton solution associated with the second Chern number developed above. Within this mathematical contest, it is possible to make a direct relationship between thermodynamics and quantum formalism. Feynman path integral give us an expression for the amplitude of probability to evolve a particle from *x*_*i*_(0) to *x*_*f*_(*t*) a time later by $< {x}_{f}|{e}^{-iHt}|{x}_{i} > ={\int }_{{x}_{i}}^{{x}_{f}}\,Dx{e}^{-iS}$, where *S* is the classical action, which could be obtained by $S={\int }_{0}^{t}\,dt^{\prime} [\frac{{p}^{2}}{2m}-V(x)]$. On the other hand, in Statistical Mechanics the partition function is defined in quite a similar form by *Z*(*β*) = *tre*^−*βH*^ where $\beta \equiv \frac{1}{{k}_{B}T}$. There is a way to change from one to the other using the Wick trick, i.e., doing the time a pure complex variable and transforming the Minkowskian space-time in a Euclidean one. Thus, we have $-iS={\int }_{0}^{-i\tau }\,d\tau ^{\prime} [\frac{{p}^{2}}{2m}-V(x)]={S}_{E}$ where *S*_*E*_ is the new Euclidean action. This fixes the concept of temperature relating it directly with the time by *τ* = ℏ*β* making $tr{e}^{-\beta H}=\int \,d{x}_{i} < {x}_{i}|{e}^{-\beta H}|{x}_{i} > =\int \,d{x}_{i}{\int }_{x\mathrm{(0)}={x}_{i}}^{x(\beta )={x}_{i}}\,Dx{e}^{-{S}_{E}}$, where we have assumed a cyclic motion *x*(0) = *x*(*β*), i.e, the particle must come back where it started after Euclidean time *τ* = ℏ*β*^22,23^. This is exactly how Berry’s phase works and justify partially its introduction as a gauge field.

On the other hand, Berry’s phase is directly related with the electric polarization ***P***, i.e., $${\rm{\Delta }}P=\frac{e}{2\pi }{\int }_{0}^{\tau }\,dt{\int }_{\frac{-\pi }{a}}^{\frac{\pi }{a}}$$$$dk{\sum }_{n\in occu}\,{f}_{n}(k)$$ being $${f}_{n}(k)=i[\frac{{\rm{\partial }}}{{\rm{\partial }}k} < {\psi }_{nk}(t)|\frac{{\rm{\partial }}}{{\rm{\partial }}t}|{\psi }_{nk}(t) > -\,\frac{{\rm{\partial }}}{{\rm{\partial }}t} < {\psi }_{nk}(t)|\frac{{\rm{\partial }}}{{\rm{\partial }}k}|{\psi }_{nk}(t) > ]$$ and under a gauge transformation of the electromagnetic potentials *A*_*μ*_ → *A*′_*μ*_ = *A*_*μ*_ + ∂_*μ*_Λ(*x*, *t*) the electron wave function transforms $$\psi \to \psi =\psi {e}^{ie\frac{{\rm{\Lambda }}}{\hslash }}$$. The above exponential function needs to be single valued while the Λ(*x*, *t*) doesn’t. Thus we can write it as $${\rm{\Lambda }}(x,t)=\frac{2\pi \mu \hslash \tau }{e\hslash \beta }=\mu \frac{2\pi \tau }{e\beta }$$ where *μ* is a winding number quantizing the temperature. This allows to find the electric potential directly related with the temperature by

7 $$V\to V^{\prime} =V+\mu \frac{2\pi }{e}{k}_{B}T$$

which means that we have transformed the electric potential into another, plus a $\mu \frac{2\pi }{e\beta }$ thermal term. This turns out to be a fundamental result: the thermal energy appears quantized by the winding number being added to one electric potential under a gauge transformation. Notice that a Chern-Simons term was necessary since it is not gauge-invariant and appears as surface actions. This allows to have, in non-Abelian Chern-Simons, the coupling constant *g*, directly associated to the temperature, related to the Chern-Simons action transforming *S*_*CS*_ → *S*′_*CS*_ = *S*_*CS*_ + 2*π*ℏ^2^*μ*/*g*^2^ where *μ* is the topological mass.

**Figure 3:**
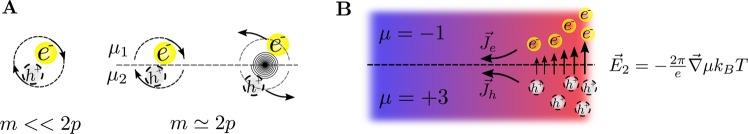
Particle-hole creation close to the topological bands. (**A**) Schematic illustration of a virtual electron-hole pair when the parameter *m* is far away from the boundary between two topological branches, that is, for example, 2*p*. When *m* is close to these value the jump in the topological mass *μ* generates a potential difference that is able to break the vacuum and to generate a real electron-hole pair representing a new mechanism to transform thermal energy into electric in solid state physics. (**B**) Representation of electron and holes creation at the hot (red) side of the TI where *m* ≈ 2*p*. Electrons and holes take place on different branches, leading to an electric field ${\overrightarrow{E}}_{2}$. Due to charge carriers are responsible to thermalize the material, this originates electron *J*_*e*_ and holes *J*_*h*_ currents where each type of carriers have different mobilities since they below to different branches.

**Figure 4:**
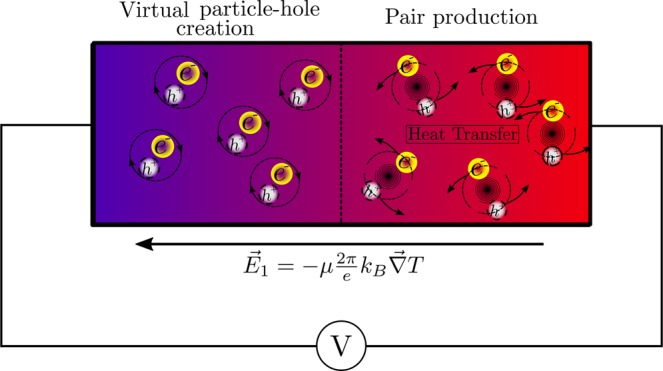
Non-Abelian topological contribution to the thermoelectric effect close to the U(1) electromagnetic surface. Charge transport through the cold (blue) side originates a potential difference *V* represented by the electric field ${\overrightarrow{E}}_{1}$. Pair production on the hot side (red) of the material leads to a local heat transfer, increasing the Seebeck coefficient.

Getting back to the topological electric potential Eq. (7), we can easy calculate the Seebeck coefficient $S=\mu \frac{2\pi }{e}{k}_{B}+\frac{2\pi }{e}\frac{\partial \mu }{\partial T}{k}_{B}T$ choosing *V*′ = 0. We can identify two contributions, the first, that comes purely from the temperature gradient in a topological branch, and the second, which takes into account the contribution due to a change in the topological index *μ*, i.e, a jump between ramifications. As we will see, the first term is equivalent to that we find when we compute the surface Seebeck coefficient while the second, doesn’t appear since there is only one ramification available on the surface. Once we have seen that the instanton solutions allow us to relate the electric potential *V* with the temperature *T* and hence the electric field ***E*** with **∇***T*, we can give a microscopic picture of how the thermal energy is invested in electricity. The main idea is that there is a creation (Fig. 3) of electron-hole Schwinger’s pairs^23–26^, provided that the electric field is big enough. Calculating the critical electric field *E*_*c*_, following Heisenberg-Euler^27^, we notice that the limit for obtaining a great number of real electron-hole pairs is

8 $${E}_{c}=\frac{{m}^{2}{v}_{F}^{3}}{e\hslash }\simeq 0.152V/nm$$

where we have considered that the energy gap is 0.21 *eV* and the Fermi velocity ${v}_{F}\simeq 6\times {10}^{5}m/s$. This provides a critical electric field of almost ten orders of magnitude lower than the critical electric field in QED and with one equivalent temperature of *ζ* = 1.74 × 10^−6^*Knm*^−1^, being these values accessible in these topological materials at so small scales as at hundredths of volt at distances of angstroms. In this way, we can rewrite the second term of the Seebeck coefficient considering that the dependence of *μ* with the temperature, as we show in Eq. (4), is represented by a Heaviside function, leading to the following expression for the Seebeck.

9 $$S=\frac{2\pi }{e}\mu {k}_{B}+\frac{2\pi }{e}\delta (\bar{p}){k}_{B}T$$

being $\bar{p}$ the different values where *μ* changes, that is, 0, ±*m*/2, ±*m*/4 and where the local increase Fig. 4, represented by the second term, can be interpreted as the contribution originated in the creation of real electron-hole pairs. This Eq. (9) of the Seebeck coefficient has two terms, the first one quantize *S* in integers due to the Chern-Simons topological mass, whereas the second corresponds to the variation of this mass respect to the temperature T. The relevant point is that the topological bands allow finding a new term in the Seebeck coefficient which can increase it depending on the value of the temperature. But what is more important, these strings of singularities separate thermally some regions from other, resulting in a temperature gradient ($\overrightarrow{\nabla }T$) which produce an electric field ${\overrightarrow{E}}_{2}$ capable to create Schwinger’s pairs whose electrons and holes have different velocities depending on the level where they are situated respect to the Fermi level. Thus, unlike what happens with metals, the effect of temperature on electrons and holes can be quite different without cancelling each other, leading to a Seebeck coefficient that would be also higher than in a semimetal. It is fundamental to observe that without the topological ramifications we would have a homogeneous crystal without thermoelectricity.

### Surface transport and dimensionless figure of merit *ZT*

**Figure 5:**
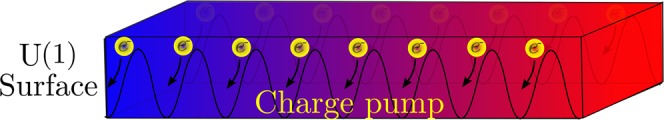
Electron charge pump in a quasi-one-dimensional surface of a TI. Electron transport takes place on the edge of the material with a quantized electrical conductivity $\sigma =\bar{n}{e}^{2}/h$.

Finally, let’s calculate the topological contribution to the surface figure of merit in TI, limiting our calculations to (2 + 1) dimensions, where the topology is determined through the first Chern number $\bar{n}$ and the winding number *n*. We start considering that, in a given direction *x* or *y*, exists a temperature gradient in our TI in such a way that eigenstates evolve adiabatically. We can define then the Seebeck coefficient through the entropy as $S=\frac{{k}_{B}}{e}(ln|\frac{{\psi }_{b}}{{\psi }_{t}}|+in\theta )$ where *ψ*_*b*_ and *ψ*_*t*_ are the final an initial states, *θ* is the angle between the states and *n* is the winding number which can only take odd integers values, otherwise the topological Seebeck coefficient would give zero. In fact, we are representing the entropy of a Riemann surface. Notice that although the von Neumann entropy would give zero considering pure states, this is not true considering that these states are in entanglement due to their global topological properties. Actually there are many local Wannier bands, i.e., ${\rm{\Psi }}(k,t)=\frac{1}{\sqrt{N}}{\sum }_{i}{\psi }_{i}\,({k}_{i},t)$, providing separate electrons which move coherently producing what is called Thouless charge pump^28^ (see Fig. 5) and relates Berry phase with the electric polarization ***P***^29–31^ (see Supplementary Information). In fact, due to the previous definitions, it is easy to see that $ln|\frac{{\psi }_{b}}{{\psi }_{t}}|\,=0$ and we can simplify the expression of the Seebeck coefficient for the TI as

10 $$S=\frac{{k}_{B}}{e}in\theta =n\frac{{k}_{B}}{e}{\int }_{C}\,i < \psi ({\bf{r}})|\nabla |\psi ({\bf{r}}) > d({\bf{r}})=\frac{\pi }{e}n\bar{n}{k}_{B}$$

being *θ* the angle directly associated to the Berry phase on the closed curve C. This leads to an expression very similar to that obtained in (4 + 1)-D, first term of Eq. (9), where the product *μw*(*U*) has been transformed into $n\bar{n}$, where *π* is the Berry phase of a non trivial material, *n* counts the number of times we complete a cycle in a system (number of singularities) and $\bar{n}$ is the first Chern number which takes into account the whole topology on the Brillouin zone for our TI.

In order to complete the calculation of the surface figure of merit, we proceed to the calculation of the electronic thermal conductivity and the electrical conductivity. For the electronic thermal conductivity, we consider the 2D density of states in the semimetal region as

11 $$D(\xi )=2\frac{1}{{\mathrm{(2}\pi )}^{2}}\int \,\delta (\xi -\hslash {v}_{F}k\mathrm{)2}\pi kdk=\frac{1}{\pi {\hslash }^{2}{v}_{F}^{2}}\xi $$

which allows us to obtain the electronic thermal conductivity *κ*_*e*_ as

12 $${\kappa }_{e}=\frac{1}{2}\frac{\partial }{\partial T}\int \,d\xi D(\xi )\xi f(\xi ){v}_{F}l=\frac{3\zeta \mathrm{(3)}}{h}{k}_{B}^{2}T$$

Being *f*(*ξ*) de Fermi-Dirac distribution function, *ζ*(3) the Riemann zeta function of dimension three (Apery’s constant) and where we have supposed a temperature dependent mean free path *l*. On the other hand, given that we have a ballistic regime for electronic transport, its conductivity *σ*_*e*_ appears given by the simple expression ${\sigma }_{e}=\bar{n}\frac{{e}^{2}}{h}$. Therefore, despite using so different expressions than the ones of the metals, where a quadratic dispersion equation is employed instead of the linear one of the semimetal, we obtain a good Wiedemann-Franz law yielded by

13 $$\frac{{\kappa }_{e}}{{\sigma }_{e}}=\frac{3\zeta \mathrm{(3)}}{\bar{n}}{(\frac{{k}_{B}}{e})}^{2}T=LT$$

where the Lorenz number $L=3\zeta \mathrm{(3)/}\bar{n}{({k}_{B}/e)}^{2}$ is one constant, as it ought to be, but divided by a Chern number $\bar{n}$ which tell us that this expression is only valid within the context of the non-trivial topological materials that we have considered. Finally, we can calculate the figure of merit *Z* for these topological insulators

14 $$Z=\frac{{\sigma }_{e}{S}^{2}}{{\kappa }_{e}}=\frac{{S}^{2}}{LT}$$

where we are not considering the phononic part of the thermal conductivity^13,32^. In this way, the dimensionless figure of merit turns out to be a simple expression

15 $$ZT={n}^{2}{\bar{n}}^{3}\frac{{\pi }^{2}}{3\zeta \mathrm{(3)}}$$

This is the extra pure topological figure of merit for the edge states, which is zero in the case of trivial topological materials. Although these conditions are quite ideal and transport constraints can diminish its efficiency under real physical features of each material, this result opens a great hope because it tells us how to improve highly the thermoelectricity associated to the topological materials. In the case of Bi_2_Te_3_^7^, for the quantum numbers equal to one we obtain a value close to the one of its present maximum, i.e. *ZT* = 2.737.

## Discussion

In summary, we have shown the relationship between topological insulators, such as the family Bi_2_Te_3_, as well as other topological related materials without time inversion protection as the Pb_1−*x*_Sn_*x*_Te, and their associated thermoelectricity. We have also seen that the second Chern number obtained for the non-Abelian *SU*(2) field leads to a thermal topological mass on a Chern-Simons action. This is equivalent to have a quantized temperature working in a kind of topological bands that we define as the ramification branches using the Riemann-Hurwitz formula on a Euclidean spacetime where instanton solutions substitute Bloch oscillating states. Physically what we have is a pumped charge between bands connected by the non-Abelian Berry phase within the insulator bulk at low temperature with an electromagnetic background field on the surface. Therefore, close to the surface we have only an Abelian *U*(1) Chern-Simons term providing us with one transformation between electric and thermal energy because we have only one kind of states. Moreover, we show that the Schwinger’s electron-hole pairs, close to the topological bands, produce an increase of the Seebeck coefficient contributing to the transformation of thermal into electric energy which is one of the key points of the model that we present in this paper. Finally, we calculate a general expression to the dimensionless figure of merit in terms of the Chern number and winding number, getting a value that coincides quite well with the one experimentally measure for the Bi_2_Te_3_, doing zero its phononic thermal contribution. It is open for future a new class of topological materials using topological indices higher than one which can cross what is considered nowadays the efficient critical value of four for the ZT figure of merit changing the physical conditions suggesting in the presented model.

## Supplementary information

Supplementary information
