# Supplementary material for: On Behind the Physics of the Thermoelectricity of Topological Insulators
Source: Sci Rep. 2019 Apr 19;9:6324. doi: 10.1038/s41598-019-42744-3 (PMC6474903; doi:10.1038/s41598-019-42744-3)
Supplement: Supplementary file 1 — Supplementary information [file 41598_2019_42744_MOESM1_ESM.pdf]

Supplementary information for
On Behind the Physics of the Thermoelectricity of Topological Insulators

Daniel Baldomir[1*] and Daniel Faílde[1]

[1]Departamento de Física Aplicada, Instituto de Investigacións Tecnolóxicas. Universidade de Santiago de Compostela E-15782 Campus Vida s/n, Santiago de Compostela, Spain

*daniel.baldomir@usc.es

S-1. Variation of the Seebeck coefficient during a complete cycle ΔS

In this section, we go further in the physical interpretation of the Seebeck coefficient in TI, calculating its variation during one cycle ($n = 1$) in the vector parameter. In this way, for an adiabatical evolution of the eigenstates we can write ΔS as follows

$$\Delta S = \frac{k_B}{2e} \int_0^{\tau} dt \int_{\frac{-\pi}{a}}^{\frac{\pi}{a}} dk \sum_{n \in occu} \frac{\partial a_n(k)}{\partial t} \tag{S1}$$

where a is the lattice constant considered in the Brillouin zone, τ is the period of time that takes a complete cycle in the parameter space, and $a_n(k)$ the Berry's connection in the k-space whose temporal derivative is taken on all the occupied states in the TI. Notice that τ will depend on the temperature gradient in the same way as it does on an external electric field whose magnitude tell us how fast the eigenstates evolution occurs. Applying Stokes we can obtain ΔS as a function of the Berry's curvature $f_n(k)$ given an associated electric polarization ΔP

$$\Delta S = \frac{k_B}{e^2} \Delta P \tag{S2}$$

where ΔP is

$$\Delta P = \frac{e}{2\pi}\int_0^{\tau} dt \int_{\frac{-\pi}{a}}^{\frac{\pi}{a}} dk \sum_{n\in occu} f_n(k) \tag{S3}$$

and the curvature $f_n(k) = i[(\frac{\partial}{\partial k} < \psi_n k(t))|\frac{\partial}{\partial t}|\psi_n k(t) > -((\frac{\partial}{\partial t} < \psi_n k(t))|\frac{\partial}{\partial k}|\psi_n k(t) >]$. Finally, the change in the Seebeck coefficient turns out to be a very simple quantity

$$\Delta S = \bar{n}\pi a \frac{k_B}{e} \tag{S4}$$

being used the simplest expression of the polarization $\Delta P = \bar{n}ae$.
